# Biomarkers for Treatment Response in Advanced Prostate Cancer

**DOI:** 10.3390/cancers13225723

**Published:** 2021-11-16

**Authors:** Samia Asif, Benjamin A. Teply

**Affiliations:** Department of Internal Medicine, Division of Hematology/Oncology, University of Nebraska Medical Center, Omaha, NE 68198-6840, USA; samia.asif@unmc.edu

**Keywords:** biomarkers, prostate cancer, predictive, prognostic

## Abstract

**Simple Summary:**

Prostate cancer is a leading cause of cancer-related death among males. Many treatments are available to manage the disease, but despite this, ultimately advanced prostate cancer is incurable and fatal. In order to improve survival and minimize side effects from these various treatments, the treatments need to be given in an optimal sequence or combination. This optimal use of therapies must be individualized, and biomarkers can be used for these decisions. Biomarkers can be useful in predicting whether a patient will respond to a treatment option and may help avoid use of therapies that are not expected to be effective. Many biomarkers are already in clinical use while many others are currently being investigated and may become part of clinical practice in future. In this review, we discuss both established and novel biomarkers with a role in management of advanced prostate cancer.

**Abstract:**

Multiple treatment options with different mechanisms of action are currently available for the management of metastatic prostate cancer. However, the optimal use of these therapies—specifically, the sequencing of therapies—is not well defined. In order to obtain the best clinical outcomes, patients need to be treated with the therapies that are most likely to provide benefit and avoid toxic therapies that are unlikely to be effective. Ideally, predictive biomarkers that allow for the selection of the therapies most likely to be of benefit would be employed for each treatment decision. In practice, biomarkers including tumor molecular sequencing, circulating tumor DNA, circulating tumor cell enumeration and androgen receptor characteristics, and tumor cell surface expression (PSMA), all may have a role in therapy selection. In this review, we define the established prognostic and predictive biomarkers for therapy in advanced prostate cancer and explore emerging biomarkers.

## 1. Introduction

Prostate cancer is the most common malignancy among males in United States, with an incidence rate of 111.3 per 100,000 men per year (2014–2018) [1]. It remains the fifth leading cause of cancer-related mortality in the United States [1]. While the majority of cases of prostate cancer are diagnosed in a localized stage and are curable, metastatic prostate cancer remains fatal.

Androgen-deprivation therapy (ADT), either via medical or surgical castration, remains the central component of the management of advanced prostate cancer [2]. Multiple treatment options are available for the management of metastatic prostate cancer beyond ADT, including chemotherapy, radiopharmaceuticals, immunotherapy, and agents targeting androgen receptor (AR) signaling. However, the optimal sequence or combination of treatments for any individual patient has been poorly defined. Given the availability of these multiple agents with different mechanisms of action, there is a need to develop biomarkers to help optimize management decisions for individual patients. The use of biomarkers to best define the most effective treatment sequence has the ability to significantly improve outcomes for patients using the already available armamentarium of therapeutics. Biomarkers, in turn, will also allow the avoidance of adverse effects from a modality that is not expected to be effective.

The National Cancer Institute (NCI) defines a biomarker as a “biological molecule found in blood, other body fluids, or tissues that is a sign of a normal or abnormal process, or of a condition or disease. A biomarker may be used to see how well the body responds to a treatment for a disease or condition” [3,4].

With the incorporation of personalized medicine and genomic sequencing in the management of prostate cancer, targetable molecular alterations were identified, as well as mechanisms of resistance that can be used to determine which available treatment is likely to be most clinically advantageous for a patient [5]. Herein, we review both established and novel biomarkers for treatment response in advanced prostate cancer.

## 2. Treatment Options for Metastatic Prostate Cancer

Androgen-deprivation therapy (ADT) is the first-line treatment for all patients with metastatic prostate cancer, including both metastatic hormone-naïve/sensitive prostate cancer (mHSPC) and metastatic castration-resistant prostate cancer (mCRPC) [6]. ADT options include bilateral orchiectomy and Luteinizing hormone-releasing hormone (LHRH) agonists or antagonists [6].

The androgen receptor (AR) is expressed on luminal prostate cells with three major functional domains: a DNA-binding domain (DBD), a ligand-binding domain (LBD) and an N-terminal domain (NTD). It is encoded for by the AR gene located on X-chromosome q11-12 [7,8]. AR is a ligand-dependent transcription factor that is activated by binding to androgenic hormones, including testosterone or dihydrotestosterone (DHT); translocation to the nucleus and the subsequent activation of downstream signals result in the growth and development of the prostate gland [9]. Prostate cancer cells express high levels of AR with excessive activation of AR signaling pathways, leading to tumor proliferation and growth [10]. As a result, the AR is an essential target in treatment of prostate cancer [11]. Nonetheless, the presence of AR is not clinically assessed prior to therapy initiation for prostate adenocarcinoma.

After the development of metastatic CRPC, ADT with an LHRH agonist or antagonist is continued and additional therapies, such as chemotherapy, secondary hormone therapy, radiopharmaceuticals, immunotherapy, or targeted therapy are initiated sequentially. Clinical trials and best supportive care are considered where appropriate.

Androgen receptor signaling inhibitors (ARSIs), including abiraterone acetate and enzalutamide, are secondary hormone therapy agents available for the management of advanced prostate cancer. Resistant metastatic prostate cancer tissues have a higher concentration of testosterone due to increased expression of enzymes for androgen synthesis such as cytochrome P450 17 alpha-hydroxylase/17,20 lyase (CYP17A1) [12,13]. Abiraterone acetate is a CYP17A1 inhibitor that is used, in combination with prednisone, for treatment of newly diagnosed mHSPC as well as for mCRPC, irrespective of prior treatment with chemotherapy [14,15]. Enzalutamide is an AR-signaling inhibitor that competes with DHT and binds to the androgen-binding site of AR, preventing the activation of AR [16]. It is effective in CRPC, having been specifically developed to overcome the overexpression of AR [17]. It is a preferred treatment option for mCRPC in patients irrespective of prior treatment with chemotherapy [18,19] and is also approved for use in mHSPC [20,21].

Among chemotherapy agents, docetaxel is approved by the Food and Drug Administration (FDA) for mCRPC, particularly for the treatment of symptomatic metastatic disease and in cases of rapid disease progression or visceral lesions [22]. Cabazitaxel is approved for mCRPC previously treated with docetaxel [23,24]. Both docetaxel and cabazitaxel inhibit microtubule depolymerization, causing cell cycle arrest [25,26]. Mitoxantrone is used for symptom palliation and results in improvements in quality of life but no improvement in overall survival (OS) [27,28]. For patients with small cell cancer of the prostate, platinum-based chemotherapy is the standard treatment, based on extrapolation from management of small cell lung cancer [29]. Platinum agents form covalent links with deoxyribonucleic acid (DNA) causing DNA damage. Apart from small cell prostate cancer, platinum-based regimens have not demonstrated a significant OS benefit in most prior studies [30]. However, a combination of carboplatin with cabazitaxel was demonstrated by Corn et al. [31] to improve progression free survival (PFS) compared to cabazitaxel alone in mCRPC in patients with features of aggressive variant prostate cancer such as visceral metastases only, small cell prostate cancer histology, and lytic bone lesions, among others [31].

Among the radiopharmaceutical therapies in clinical use, Radium-223 is an alpha-emitting radioactive agent that provides survival benefit in patients with symptomatic bone metastases [32]. Beta-emitting pharmaceutics such as Strontium-89 (^89^Sr) or Samarium-153 (^153^Sm) are utilized for the palliation of painful bone lesions without a survival benefit [33]. Lutetium-177-PSMA radionuclide therapy is an emerging treatment strategy for mCRPC following progression on prior AR signaling inhibitors and one or two taxanes, and utilizes Lutetium-177 (^177^Lu), a medium energy *beta-*emitter [34]. Prostate-specific membrane antigen (PSMA) is a prostate cell surface receptor that is overexpressed in metastatic tumors [35]. The internalization of proteins bound to PSMA into endosomes allows PSMA-labelled isotopes to be concentrated within prostate cancer cells [36]. PSMA-targeted radioimmunotherapy involves the binding of a radioligand with an antibody to PSMA in order to increase the dose delivered to tumor cells with the sparing of normal tissue [37]. Different PSMA peptides and anti-PSMA antibodies are labelled with ^177^Lu [38,39].

Olaparib and rucaparib are oral inhibitors of Poly (ADP-ribose) polymerase (PARP). Olaparib is approved for use in patients with mCRPC, previously treated with enzalutamide or abiraterone, who have deleterious germline or somatic mutations in genes involved in homologous recombination repair, i.e., a mutation in at-least one of 14 genes: *BRCA1*, *BRCA2*, *ATM*, *CHEK1*, *CHEK2*, *PALB2*, *RAD51B*, *RAD51C*, *RAD51D*, *RAD54L*, *BARD1*, *BRIP1*, *CDK12*, or *FANCL* [40,41]. Rucaparib is approved for mCRPC patients with pathogenic alterations in *BRCA1* or *BRCA2* and previously treated with AR-directed therapy and taxane-based chemotherapy [42]. Despite FDA approval, the optimal set of predictive biomarkers for response to PARP inhibition in prostate cancer remains to be defined.

Immune checkpoint inhibitors (ICI) in clinical use include monoclonal antibodies targeting Programmed-death receptor-1 (PD1), Programmed-death ligand-1 (PDL-1) and cytotoxic T-lymphocyte-associated protein-4 (CTLA-4). Despite extensive testing in prostate cancer, these remain FDA-approved only for a very small percentage of patients who would qualify under the tissue-agnostic approvals for ICIs. Pembrolizumab is approved for use in patients with mCRPC with microsatellite instability (MSI) and mismatch repair deficiency (dMMR) [43], for example.

Table 1 summarizes the mechanism of action and clinical indications of therapeutic agents used in the management of metastatic prostate cancer that are discussed in this review.

## 3. Clinical Prognostic Biomarkers

Prognostic markers offer information regarding a patient’s overall outcome, such as risk for recurrence after receiving standard treatment. This can help in the selection of patients for treatment but does not predict response to a particular therapy [45]. Multiple prognostic factors for survival in advanced prostate cancer have been evaluated, including hemoglobin, erythrocyte sedimentation rate (ESR), alkaline phosphatase (ALP) and lactate dehydrogenase (LDH) [46], though, given associated patient factors such as malnutrition- and cancer-related complications, these may not entirely reflect cancer-specific survival (CSS). Prognostic markers that are currently available for metastatic prostate cancer are discussed below.

### 3.1. Prostate-Specific Antigen (PSA) Nadir and Kinetics

PSA as a biomarker is utilized in prostate cancer screening, risk stratification for recurrence and, subsequently, for monitoring of therapy [47]. A retrospective review of patients receiving primary ADT for metastatic prostate cancer by Sasaki et al. [46] indicated that a lower PSA nadir with ADT and a longer time to PSA nadir (>9 months) was associated with improved overall survival in prostate cancer patients with bony metastases [46].

Serial determinations of PSA are used to monitor response to therapy [47]. For patients with mHSPC, data from Southwest Oncology Group (SWOG) Trial 9346 suggest a role for PSA level after 7 months of ADT as a predictor of survival [48]. Achieving a PSA of 4 ng/mL or less after 7 months of ADT may be a predictor of risk of death in mHSPC, with patients with a PSA of 0.2 ng/mL or less having the greatest survival advantage [48]. More precisely, the risk of death in group with a PSA of ≤4 ng/mL at 7 months was less than one-third of group with a PSA > 4 ng/mL. Those with a PSA of ≤0.2 ng/mL had one-fifth the risk of death compared to those with PSA > 4 ng/mL and significantly better survival than those with a PSA of >0.2–4 ng/mL. This may allow patients who are less likely to do well on ADT to be recognized sooner, yielding a window for earlier intervention with additional therapies before developing overt castration-resistance.

In patients with CRPC but no distant metastases seen on conventional imaging, PSA doubling time (PSADT) greater than 10 months predicted a relatively indolent disease course [49]. However, patients with PSADT < 10 months are at high risk for the development of metastases. This clinical biomarker of PSADT is currently employed to select patients with non-metastatic CRPC for additional therapy with a potent antiandrogen such as enzalutamide, apalutamide, or darolutamide [50,51,52].

### 3.2. Baseline Serum Testosterone

Higher serum testosterone levels prior to ADT initiation in mCRPC are associated with increased survival. A study by Chodak et al. [53] suggested a 2-year survival rate of 67% for men with serum testosterone levels of 8.6 nmol/L and above vs. 30% in a group with testosterone levels below 8.6 nmol/L [53]. It logically follows that prostate cancers that are progressing despite lower serum testosterone have already adapted in-part to a low testosterone state and, therefore, will be less sensitive to further androgen deprivation.

### 3.3. HSD3B1 Genotype

The enzyme 3-beta-hydroxysteroid dehydrogenase-1 (HSD3B1) catalyzes the conversion of dehydroepiandrosterone (DHEA) to testosterone and DHT in prostatic tissue; this is encoded for by the gene *HSD3B1. HSD3B1(1245A)* is an adrenal-restrictive allele that leads to the production of an enzyme that is more rapidly degraded, limiting conversion of DHEA to DHT. On the other hand, *HSD3B1(1245C)* is an adrenal-permissive allele, encoding for a more stable enzyme and resulting in more DHT production [54].

Hearn et al. [55] evaluated whether inheritance of *HSD3B1(1245C)* was associated with worse clinical outcomes in patients with mHSPC who had been randomized to receive ADT with or without docetaxel as part of the E3805 Chemo-Hormonal Therapy vs. Androgen Ablation Randomized Trial for Extensive Disease in Prostate Cancer (CHAARTED) trial [55]. Patients received ADT with or without six cycles of docetaxel. Metastatic disease was considered as “high volume” when visceral metastases or four or more bone metastases (with at least one lesion beyond pelvis and vertebral bodies) were present. Disease was considered “low volume” otherwise. In patients with low volume disease, freedom from CRPC at 2 years was significantly lower in patients with adrenal-permissive genotypes (inheritance of at least one copy of *HSD3B1 (1245C)* compared to adrenal-restrictive genotypes (51.0% vs. 70.5%). Similarly, OS at 5 years was worse in those with *HSD3B1(1245C):* 57.5% vs. 70.8%. In those with high-volume disease, no difference was noted in terms of freedom from CRPC at 2 years or OS at 5 years between the two genotypes [55]. A difference in benefit derived from treatment with docetaxel based on *HSD3B1* genotype was not seen.

Hence, the *HSD3B1* genotype may serve as a prognostic marker for patients with mHSPC with low volume disease and as a predictive marker for response to ADT in this population, but not as a predictive marker for treatment benefit with docetaxel. The association of *HSD3B1(1245C)* with a shorter Progression free survival (PFS) on ADT in patients with mHSPC has been demonstrated in other studies as well [56,57].

Abiraterone is metabolized by 3-beta-hydroxysteroid dehydrogenase-1 (HSD3B1) into steroidal metabolites, including 3-keto-5alpha-abiraterone. This metabolite has been noted to stimulate AR and promote tumor progression in animal models. Alyamani and colleagues [58] studied the metabolism of Abiraterone in healthy volunteers with the *HSD3B1(1245C)* genotype and concluded that there was an increased production of 3-keto-5alpha-abiraterone in those with more copies of *HSD3B1(1245C)* [58]. The clinical impact of increased synthesis of this metabolite is yet to be elucidated but the authors postulate that these patients may benefit from the use of a nonsteroidal CYP17A1 inhibitor such as ketoconazole in place of Abiraterone [58,59].

### 3.4. Neutrophil-to-Lymphocyte Ratio (NLR)

The neutrophil-to-lymphocyte ratio is an easily calculated biomarker that has been studied as a prognostic factor in multiple solid malignancies including prostate cancer. Prior studies suggested that a higher pre-treatment NLR (using a cut-off 3.76) predicts a worse OS in patients treated with Abiraterone [60]. However, a change in NLR, either an increase or a decrease, from treatment baseline was not shown to be indicative of lack of response to treatment with Abiraterone in a study by Loubersac and colleagues [61] and should not be used to guide changes in therapy [61]. Subsequently, Kumano et al. [62] noted that in patients with CRPC receiving Enzalutamide, a higher pre-treatment NLR (using a cut-off 2.14) was associated with a worse OS and cancer-specific survival than those in the lower NLR group [62].

A retrospective study by Koo and colleagues [63] used a pretreatment NLR cut-off of 2.5 in patients who had received docetaxel before or after AR-directed therapy (either Abiraterone or Enzalutamide). Patients with an NLR of 2.5 or greater had lower 2-year cancer-specific survival and 1-year radiographic PFS (rPFS) compared to those with NLR below 2.5 [63]. Additionally, for patients with NLR below 2.5, better cancer-specific survival and rPFS was seen in patients who received docetaxel followed by AR inhibitors rather than vice versa. Sequencing of AR-inhibitors and docetaxel did not affect rPFS or CSS in patients with an NLR of 2.5 or above [63]. This suggests a potential use for NLR in guiding the sequencing of therapy in CRPC in patients where the NLR is below 2.5 and may have roles both as a prognostic and predictive biomarker that need to be investigated further.

### 3.5. Circulating Tumor Cells (CTC) Enumeration

The detection of circulating tumor cells (CTCs) has been an emerging biomarker in solid malignancies including prostate cancer [64,65]. CTC assessment using CellSearch TM (Janssen Diagnostics, Raritan, Somerset County, NJ, USA) has been approved by the FDA as a prognostic tool in prostate cancer [66,67].

Mejean et al. [68] tested peripheral blood from prostate cancer patients and controls using a reverse-transcriptase polymerase chain reaction (RT-PCR) that targeted PSA mRNA. Detectable CTCs were noted in 33% of cancer patients compared to only 2% in the control group. At follow up ranging from 4 to 49 months, the detection of CTCs was shown to be associated with increased risk of developing metastases and relapse [68].

In a study by Kantoff et al. [69] the detection of CTCs using RT-PCR for PSA was shown to predict poorer survival in patients with CRPC. Median survival in those with detectable PSA transcripts in peripheral blood was 13 months compared to 18 months without detectable PSA transcripts [69].

According to De Bono et al. [66], in mCRPC patients with disease progression and who were starting a new treatment, a higher or “unfavorable” CTC count (5 or greater CTC/7.5 mL) both pre-treatment and post-treatment (2 to 5 weeks after initiating new treatment) predicted a shorter OS compared to a lower or “favorable” CTC count (less than 5/7.5 mL). If the post-treatment CTC count converted from unfavorable to favorable, the OS improved (6.8 to 21.3 months). If the post-treatment CTC count converted from favorable to unfavorable, then the OS worsened (from greater than 26 months to 9.3 months) [66].

Based on these findings, the use of CTC may be used to monitor the effects of different treatments in CRPC when measured before and after treatment.

### 3.6. Total Alkaline Phosphatase (tALP)

For patients receiving Radium-223, a normal pre-treatment total ALP is associated with longer OS than with an elevated tALP. A reduction of 10% or greater in an elevated baseline tALP at 4 weeks or beyond from treatment is also associated with improved OS [70].

Similarly, changes in ALP in patients with osseous metastatic disease who received docetaxel or mitoxantrone were studied by Sonpavde and colleagues [71]. All patients had elevated ALP at baseline. Patients with normalization of ALP at day 90 of treatment had better median OS compared to those without normalization (18.8 vs. 13.4 months) [71]. Patients with a higher baseline ALP were less likely to have normalization with treatment. An increase in ALP prior to day 90 was associated with poorer OS compared to that seen without an ALP increase (10.5 months vs. 15.3 months). This prognostic role of ALP was seen in all treatment arms [71]. Nonetheless, tALP is not specifically used to clinically select patients for radium-223 at this time.

## 4. Clinical Predictive Biomarkers

Predictive markers provide information regarding benefit from a particular treatment or the difference in outcomes between two or more interventions [45]. Predictive markers available in the management of metastatic prostate cancer are discussed below.

Table 2 summarizes predictive and prognostic biomarkers that are available or emerging in the management of prostate cancer for each of the different treatment strategies.

### 4.1. AR Splice Variant 7

Patients with AR-V7 mRNA detected in circulating tumor cells (CTCs) derive greater survival benefit, both in terms of overall survival and progression free survival with taxane therapy, compared to those with abiraterone or enzalutamide [74,92]. AR-V7 results in a truncated isoform of AR that lacks the ligand-binding C-terminal that is the therapeutic target of AR-directed therapies, resulting in resistance to ADT and AR-targeted agents but not to taxanes [93]. Therefore, CTC based AR-V7 detection can serve as a predictive marker for lack of response to Enzalutamide or abiraterone [74,75].

On the other hand, Tagawa et al. [79] evaluated the response to taxane therapy in patients with expression of the AR-V7-truncated isoform and an exon-skipping AR variant, AR^v567es^, compared to those with a full-length AR [79]. The hinge domain that mediates binding of microtubules is absent from AR-V7 but is present in AR^v567es^, although with reduced affinity for microtubules [79]. Patients without either variant were noted to have superior PFS (16.6 months) when treated with taxane therapy. Patients with AR^v567es^ had better PFS compared to those with AR-V7 (11.2 months vs. 8.5 months). This suggests that absence of AR splice variants is associated with superior PFS in patients with mCRPC when treated with cabazitaxel or docetaxel [79].

A recent study by Li et al. [80] suggests that mHSPC patients with AR-V7 expression have a shorter PFS (22 vs. 10 months) compared to patients without AR-V7 expression. AR-V7 expression appears to be additionally associated with a poorer OS [80].

### 4.2. Circulating Cell-Free DNA (cf-DNA) or Circulating Tumor-DNA (ct-DNA)

Ct-DNA or cf-DNA are small nuclear acid fragments present in the bloodstream secondary to either the apoptosis and necrosis of primary tumor cells or the release of intact tumor cells in the bloodstream that subsequently undergo lysis [94]. cf-DNA carries tumor-related genetic and epigenetic changes that have a role in cancer progression and treatment resistance [95].

In November 2020, the FDA approved the next-generation sequencing (NGS)-based FoundationOne Liquid CDx test that uses cf-DNA isolated from plasma of cancer patients for use in mCRPC to identify mutations on the *BRCA1*, *BRCA2* and *ATM* genes [76]. Azad et al. [77] detected AR gene aberrations that confer resistance to enzalutamide and Abiraterone including AR amplification and AR-exon 8 mutations in cf-DNA derived from mCRPC patients [77]. Similarly, Wyatt et al. [78] studied cf-DNA in patients with mCRPC and noted that worse PFS was seen if an AR amplification, two or more mutations in the *AR* gene and a loss of *RB1* was seen [78]. At progression, cf-DNA analysis showed mutations or changes in copy numbers, including actionable mutations in the phosphatidylinositol 3-kinase (PI3K) pathway and DNA-repair genes [78]. Limitations in the use of CTC and cf-DNA include procedural difficulty in isolating CTCs and subsequent nucleic acid extraction and a low volume of CTCs and cf-DNA present in the bloodstream for detection [67]. The data obtained thus far, however, do not suggest a clinical predictive biomarker role for cf-DNA for first-line ARSIs.

### 4.3. Serum Testosterone Levels

For patients receiving AR-targeted therapy, pre-treatment serum testosterone levels potentially have a predictive role. According to Hashimoto et al. [72], patients with testosterone levels between 5 and 50 ng/dL had longer PSA-PFS than those with levels below 5 ng/dL. For patients with testosterone below 5 ng/dL, the rate of PSA response was higher in patients treated with Abiraterone compared to enzalutamide [72].

In another study involving mCRPC patients receiving enzalutamide, a superior PFS was seen in patients with pre-treatment testosterone levels greater than 0.05 ng/mL compared to levels below 0.05 ng/mL. While OS showed a trend towards superiority in the higher testosterone group, the results were not statistically significant. For patients receiving Abiraterone, no difference in PFS or OS was seen in patients with testosterone levels above or below 0.05 ng/mL [73]. Conversely, for mCRPC patients treated with docetaxel, PFS was worse in patients with testosterone levels above 0.05 ng/mL [73]. Hence, pre-treatment serum testosterone levels may have predictive value in the selection of AR targeted therapy versus chemotherapy in patients with mCRPC.

### 4.4. Homologous Recombination Repair (HRR) Mutations

The BRCA1 and 2 proteins play a role in double-stranded DNA (dsDNA) break repair via the homologous recombination repair process (HRR), a conservative repair mechanism that restores the original DNA sequence at the site of DNA damage. Other proteins affecting HRR include ATM, CHEK1, CHEK2, RAD51, PALB2, and FANCA [96]. In settings involving an HRR deficiency due to defects in BRCA1 or two or other proteins involved in this pathway, a non-conservative DNA repair processes come into play resulting in DNA alterations that can increase cancer risk [97]. In mCRPC patients, up to 15–20% patients have genetic changes causing HRR deficiency [98].

The enzymes Poly (ADP-ribose) polymerase (PARP)-1 and PARP2 participate in DNA damage repair by binding to sites of single-stranded DNA (ssDNA) breaks and recruiting DNA-repair proteins. PARP inhibitors prevent correction of these ssDNA breaks, stalling the DNA replication fork and generating dsDNA breaks. In the presence of an underlying genetic deficiency in DNA damage repair mechanisms or an additional cytotoxic drug, PARP inhibitors result in DNA damage accumulation that drives their anti-neoplastic effect [97,99].

In a phase 3 trial, de Bono et al. [89] studied olaparib in patients with mCRPC who had an alteration in one of 15 genes involved in homologous recombination repair [89]. These included deleterious or suspected deleterious changes in *BRCA1*, *BRCA2*, *ATM*, *PPP2R2A*, *CHEK1*, *CHEK2*, *PALB2*, *RAD51B*, *RAD51C*, *RAD51D*, *RAD54L*, *BARD1*, *BRIP1*, *CDK12*, or *FANCL* identified on a tissue biopsy sample of either primary or metastatic disease. Cohort A included patients with at least one alteration in *BRCA1*, *BRCA2*, or *ATM*. Cohort B included patients with alterations in any of the 12 remaining genes. In each cohort, patients were randomized to receive olaparib versus either enzalutamide or abiraterone. In cohort A, patients receiving olaparib had a longer PFS (7.4 months vs. 3.6 months), a longer OS (18.5 months vs. 15.1 months), and a higher Objective Response rate (ORR) (33% vs. 2%) than the control arm. When the combined study population was evaluated, i.e., both cohort A and B, PFS was longer (5.8 months vs. 3.5 months), ORR was higher (22% vs. 4%), and median OS was prolonged (17.5 months vs. 14.3 months) in patients receiving olaparib [89]. Currently, the FDA has approved testing for either germline or somatic deleterious mutations in BRCA1 and 2 from tumor or liquid biopsy [38].

HRR deficiency may also be predictive of response to immune checkpoint inhibitors (ICIs) as discussed later in this review.

Mota et al. [82] evaluated the response to platinum-based chemotherapy in patients with alterations in DNA damage repair genes, including *BRCA1*, *BRCA2*, *ATM*, *PALB2*, *CDK12*, and *FANCA.* In 16 patients with taxane-refractory disease who had a mutated DNA damage repair gene, a greater likelihood of a 50% decline in PSA on platinum therapy than those without such a gene alteration was observed. In this study, 4 of 6 patients with *BRCA2* mutation achieved a 50% decrease in PSA; patients with *PALB2*, *FANCA*, and *CDK12* alterations experienced similar responses. None of the patients with a deleterious *ATM* mutation (*n* = 4), however, showed any response to platinum agents. However, OS was not significantly different between patients with and without alterations in DNA damage repair genes. Interestingly, among the patients who had progressed on prior PARP inhibitors, 6 of 7 experienced a decline in PSA [82]. Alterations in DNA damage repair genes may be predictive of response to platinum agents.

### 4.5. Tumor Mutational Burden (TMB) and PDL-1 Expression

Ipilimumab, an anti-CTLA-4 antibody, increases T-cells infiltration of tumor. This results in up-regulation of PD-1/PDL-1 pathways that are immune-inhibitory, causing T-cell suppression [100]. This may be overcome by using it in combination with Nivolumab, an anti-PD1 agent. The phase II CheckMate 650 study evaluated the nivolumab/ipilimumab combination in mCRPC [91,101]. Compared to those with below median TMB, patients with a higher TMB (i.e., above median) experienced higher objective response rates (ORR) (50.0% vs. 5.9%) and OS (19.0 vs. 10.1 months) [91]. Patients with homologous recombination repair deficiency had higher ORR and median OS compared to those without such deficiency (50.0% vs. 22.6%; median survival not reached vs. 19.0 months, respectively) [91]. Patients with DNA damage repair mutations had higher ORR (36.4% vs. 23.1%) and longer median OS (not reached vs. 19.0 months) compared to those without these mutations [91]. Better ORR (26% vs. 10%) was seen in patients with PD-L1 greater than or equal to 1% compared to PD-L1 less than 1% [101]. This study suggests that PDL1 greater or equal to 1%, HRR deficiency, mutations in DNA damage repair genes, and high TMB may all predict response to ICI in mCRPC [102,103].

However, KEYNOTE-199 suggested pembrolizumab monotherapy may benefit a small number of patients with mCRPC irrespective of Combined Positive Score (CPS) [104]. A phase II study by Graff et al. [105] evaluated the combination of Pembrolizumab with enzalutamide; a durable response was seen in 18% (*n* = 5/28), irrespective of PDL-1 expression [105]. PD-L1 expression may not be a useful marker of response to ICI monotherapy. Further testing is ongoing to identify a subpopulation and biomarker to identify potential candidates for ICI in prostate cancer.

### 4.6. Microsatellite Instability (MSI) and Mismatch Repair Deficiency (dMMR)

Tumors with mismatch repair deficiency (dMMR) carry a high number of somatic mutations, up to 10 to 1000 times greater than mismatch repair proficient tumors [102,106]. This includes mutations in repetitive DNA sequences called microsatellites, resulting in high levels of microsatellite instability (MSI-H) [103]. The genes involved in mismatch repair include *MLH*, *MSH2*, *MSH6*, and *PMS2*; a biallelic defect in these genes causes mismatch repair deficiency [103]. Tumors with dMMR have a higher number of infiltrating lymphocytes and tumor cells can express PD-L1 on their membranes. Hence, tumors with dMMR/MSI-H are expected to have a robust anti-tumor response to immune check point blockade [102,107], suggesting that MSI-H can serve as a biomarker for PD-1 blockade. Pembrolizumab may be used in mCRPC patients with dMMR/MSI-H [108]. The prevalence of dMMR in mCRPC is 2–5% [106].

## 5. Experimental Biomarkers

A multitude of biomarkers have emerged that may become established in the future as predictive markers of response to different treatments in metastatic prostate cancer. Some of these novel biomarkers are highlighted below.

### 5.1. ERG and SOX9

The E26 transformation-specific (ETS)-related gene (*ERG*) is an overexpressed oncogene in prostate cancer. This overexpression results from a *TMPRSS2-ERG* fusion between the androgen-driven promoter of *TMPRSS2* gene and the coding region of *ERG* [109]. *ERG* binds to microtubules and inhibits the drug–target interaction that results in docetaxel resistance. SRY-related HMG box (SOX)-9 is a downstream *ERG* effector [81]. Immunohistochemistry that is positive on tissue biopsy samples for ERG and SOX9 correlates with a lower PSA response, PFS, and OS following docetaxel treatment in patients with mCRPC [81] and may help decide which patients are less likely to benefit from chemotherapy. Mellado et al. [110] evaluated *TMPRSS2-ERG* expression in the peripheral blood of patients with mCRPC using quantitative RT-PCR. In patients treated with docetaxel, those with *TMPRSS2-ERG* expression experienced a lower PSA-response rate and a lower PSA reduction compared to those negative for *TMPRSS2-ERG* expression. Additionally, *TMPRSS2-ERG* expression correlated with a lower PSA-PFS, clinical/radiological PFS and OS in patients treated with docetaxel. In this study, only five patients treated with cabazitaxel had *TMPRSS2-ERG* expression. These patients had a poorer PSA-PFS and a trend towards poorer OS and clinical/radiographic PFS. A larger number of patients would be required to define the association between *TMPRSS2-ERG* and cabazitaxel response [110]. *ERG* fusion status may be a biomarker of docetaxel resistance.

### 5.2. SLFN11 Expression

Conteduca et al. [83] investigated whether the expression of DNA/RNA helicase Schlafen family member-11 (SLFN11) affected outcomes in mCRPC patients who were treated with platinum-based chemotherapy. Expression of SLFN11 was assessed either in CTCs or biopsy tissue. In patients with overexpression of SLFN11, a longer radiographic PFS and a PSA decline of 50% or greater was seen although no difference in OS was noted [83]. SLFN11 expression may have a role in selecting patients who may benefit from platinum-based chemotherapy.

### 5.3. Bone Scan Index (BSI)

The bone scan index has been studied as a prognostic indicator of OS. A retrospective analysis by Fosbol et al. [84] suggested mCRPC patients with a lower BSI (5 or less) had better OS compared to patients higher BSI (greater than 5). Patients with a higher BSI at baseline also experienced greater hematologic toxicity from Radium-223 [84]. In a study by Naito et al. [85], patients receiving Radium-223 were categorized into two groups based on whether they achieved a decrease in BSI of at least one point. Patients with a decrease in BSI achieved a longer OS [85].

### 5.4. Bone Metabolic Markers

Bone metabolic markers (BMM) may have a role as predictive and prognostic indicators in mCRPC with bone metastases. Lara et al. [86] evaluated bone metabolic markers in patients who were enrolled in SWOG 0421 trial. This trial had previously concluded that addition of Atrasentan, an endothelin-A antagonist and a bone targeting agent, to docetaxel did not improve OS or PFS [111]. Markers of bone resorption, including N-telopeptide (NTx) and pyridinoline (PYD), and markers of bone formation, including C-terminal of type-1 collagen propeptide (CICP) and bone-specific alkaline phosphatase (BAP), were measured prior to treatment and then serially. Elevated levels at baseline were associated with poor OS. Increasing levels of BMMs after week nine of treatment were associated with poor OS, while a serial decrease in levels of urine N-telopeptide and serum BAP suggested better OS. Interestingly, for patients with a higher baseline CICP and BAP (upper 25th percentile), an improved OS was seen in the Atrasentan arm [86], suggesting these bone metabolic markers may have a predictive role in determining which patients may benefit from bone-targeted therapies.

Agarwal and colleagues [87] studied changes in serum bone metabolic markers (including N-telopeptide, bone-specific alkaline phosphatase, C-telopeptide (CTP), N-terminal propeptide of collagen-1 (P1NP) and pyridinoline) at baseline and at 6 months in patients receiving enzalutamide with or without Radium-223 [87]. A statistically significant decrease in Bone metabolic markers including N-telopeptide, BAP and P1NP was noted in patients receiving Radium-223. A decrease in bone metabolic markers also correlated with better clinical outcomes, including improved PFS and median OS in the arm receiving Radium-223 [87]. These findings suggest a possible predictive role for bone metabolic markers in patients treated with Radium-223.

### 5.5. Prostate-Specific Membrane Antigen (PSMA) Expression

Prostate-specific membrane antigen (PSMA) expression in primary tumor is not predictive of metastatic disease, but a higher expression is associated with a more aggressive tumor, particularly in CRPC [35].

Not all patients with mCRPC will have a good treatment response to ^177^Lu-PSMA, where different PSMA peptides and anti-PSMA antibodies are labelled with ^177^Lu [38,112]. A Gallium-68 (^68^Ga)-PSMA-11 positron emission tomography (PET) was studied as a predictor of response to ^177^Lu-PSMA-617 treatment in a phase II trial [88]. All patients underwent a ^68^Ga-PSMA-11 PET/computed tomography (CT) and a mean (SUV_mean_) and maximum (SUV_max_) standardized uptake value (SUV) was noted. Higher intensity of PSMA-SUV_mean_ and PSMA-SUV_max_ prior to administration of ^177^Lu-PSMA-617 showed strong correlation with treatment response. In responders, the SUV_max_ was 44 ± 15 compared to 17 ± 9 in non-responders; the SUV_mean_ was 10 ± 4 vs. 6 ± 4, respectively.

For patients with SUV_max_ values of less than 15 on PSMA-PET, no biochemical treatment response to ^177^Lu-PSMA-617 was observed. The volume of metastatic disease or the nature of the site of metastases did not predict treatment response [88]. A ^68^Ga-PSMA-11 PET/CT may allow the selection of patients expected to respond best to Lutetium-177-PSMA radionuclide therapy in the future.

### 5.6. PTEN Loss

The deletion or mutation of phosphatase and tensin homologue *(PTEN*), a tumor suppressor gene, is seen in approximately 50% of CRPC [113]. Inactivation of *PTEN* is associated with castration resistance, prostate cancer-specific death and metastases [113,114]. Consequently, *PTEN* loss or inactivation is considered a biomarker for aggressive prostate cancer [90]. The loss of PTEN phosphatase function results in the activation of the phosphatidylinositol 3-kinase (PI3K)/AKT signaling pathway, which in turn promotes tumor growth and proliferation [115].

*PTEN* loss is associated with reduced response to AR-targeted agents such as abiraterone [116]. Ipatasertib is an investigational agent that is being developed for prostate cancer, likely for a biomarker-selected patient population. A phase III trial (IPATential150) evaluated the role of ipatasertib, an AKT inhibitor, in combination with Abiraterone/prednisolone in mCRPC [44]. *PTEN* loss was assessed by immunohistochemistry. Compared to the placebo-Abiraterone arm, patients in the combination arm had longer median radiographical PFS, a non-statistically significant longer PFS, and a higher ORR. OS data were still immature at the time of data cut-off. *PTEN* loss may predict improved clinical outcomes with the ipatasertib/abiraterone combination in mCRPC [44].

## 6. Conclusions

The management of advanced prostate cancer requires individualized treatment paradigms and sequences based upon the patient’s clinical status, disease characteristics, genomics, and available biomarkers. In order to maximize the outcome for patients, they need to be treated early with therapies with the highest chance for response and benefit. Patients also need to avoid trials of ineffective therapies given the multiple other treatment agents available. While some predictive biomarkers, especially for tumor genomics for targeted therapy and AR-V7 status for chemotherapy, are already deployed in the clinic, further development is required to truly guide therapy selection through the disease course.

## Figures and Tables

**Table 1:** Therapeutic agents for metastatic prostate cancer. (References in parenthesis).

Treatment Agent	Mechanism of Action	Clinical Indication
Androgen-deprivation therapy [6]	Decreased activation of AR and downstream signaling pathways	First-line treatment for both hormone-sensitive and hormone-resistant metastatic prostate cancer
Androgen receptor signaling inhibitors (ARSI)		
Abiraterone acetate [14,15]	Inhibition of androgen biosynthesis by inhibiting enzyme 17-alpha-hydoxylase/C17,20-lyase	mHSPC and mCRPC, before or after Docetaxel
Enzalutamide [18,19,21]	Androgen receptor antagonism	mHSPC and mCRPC, before or after Docetaxel
Chemotherapy		
Docetaxel [22]	Tubulin binding taxane, inhibiting microtubular depolymerization	mCRPC, including symptomatic metastatic disease, visceral metastases and rapid disease progression
Cabazitaxel [23,24]	Tubulin binding taxane, inhibiting microtubular depolymerization	Symptomatic mCRPC, second-line therapy after progression on docetaxel
Carboplatin [29,31]	Formation of covalent link with DNA causing DNA damage	Small cell or neuroendocrine prostate cancer; with cabazitaxel in aggressive variant mCRPC
Radiopharmaceutical therapy		
Radium-223 [32]	Alpha-particle emitting radioactive agent	Symptomatic bone metastases without visceral metastases
Lutetium-177 PSMA radionuclide therapy [34]	PSMA peptides and anti-PSMA antibodies labelled with beta-emitting radioactive agent	Emerging therapy for mCRPC if PSMA-PET/CT positive disease, after ARSIs and one or two taxanes
Targeted therapy		
Olaparib [40,41]	Poly (ADP-ribose) polymerase inhibitor	Second-line treatment after abiraterone or enzalutamide (with or without prior taxane) for mCRPC with homologous recombination repair mutations
Rucaparib [42]	Poly (ADP-ribose) polymerase inhibitor	mCRPC with pathogenic *BRCA1* or *BRCA2* mutation and prior treatment with ARSIs and a taxane
Ipatasertib [44]	AKT inhibitor	Emerging therapy; mCRPC with *PTEN* loss
Immunotherapy		
Pembrolizumab [43]	Anti-PD1 antibody	MSI-high or MMR-deficient mCRPC, with disease progression on at least one prior systemic therapy

Abbreviations: androgen receptor signaling inhibitors (ARSIs), V-akt murine thymoma viral oncogene homolog (AKT), anti-programmed-death receptor-1 (Anti-PD1), androgen receptor (AR), metastatic castration-resistant prostate cancer (mCRPC), metastatic hormone-sensitive prostate cancer (mHSPC), phosphatase and tensin homologue (PTEN), prostate-specific membrane antigen (PSMA), positron emission tomography/computed tomography (PET/CT).

**Table 2:** Biomarkers in management of metastatic prostate cancer based on treatment strategy. (References in parenthesis).

Intervention	Biomarker	Clinical Utility
Androgen-deprivation therapy (ADT)	Prostate-specific antigen (PSA) [48,49]	Predictive/Prognostic
	Serum Testosterone [53]	Prognostic
	HSD3B1 genotype [55]	Predictive/Prognostic
Androgen receptor signaling inhibitors (ARSIs)	Neutrophil-to-lymphocyte ratio [60,62,63]	Predictive/Prognostic
	Serum testosterone [72,73]	Predictive
	Circulating tumor cells (CTC) enumeration [66,69]	Prognostic
	AR splice variant 7 [74,75]	Predictive
	Circulating cell-free DNA or tumor-DNA [76,77,78]	Predictive
Chemotherapy	AR splice variant 7 [74,79,80]	Predictive
	*ERG*/SOX9 [81]	Predictive
	DNA damage repair gene alterations [82]	Predictive
	SLFN11 expression [83]	Predictive
Radiopharmaceutical therapy	Total alkaline phosphatase [70]	Prognostic
	Bone Scan Index (BSI) [84,85]	Prognostic
	Bone metabolic markers [86,87]	Predictive/Prognostic
	Prostate-specific membrane antigen (PSMA) expression [35,88]	Predictive/Prognostic
Targeted therapy	Homologous recombination repair (HRR) mutations [40,89]	Predictive
	*PTEN* loss [44,90]	Predictive/Prognostic
Immunotherapy	Microsatellite instability (MSI) and mismatch repair deficiency (dMMR) [43]	Predictive
	Homologous recombination repair (HRR) mutations [91]	Predictive
	Tumor mutational burden (TMB) [91]	Predictive
	PDL-1-expression [91]	Predictive

Abbreviations: androgen receptor (AR), E26 transformation-specific (ETS)-related gene (*ERG)*, 3-beta-hydroxysteroid dehydrogenase-1 (HSD3B1), programmed-death ligand-1 (PDL-1). Phosphatase and tensin homologue (*PTEN*), DNA/RNA helicase Schlafen family member-11 (SLFN11), SRY-related HMG box-9 (SOX9).

## Abbreviations

ADT	Androgen-deprivation therapy
AKT	V-akt murine thymoma viral oncogene homolog
ALP	Alkaline phosphatase
AR	Androgen receptor
ARSIs	Androgen receptor signaling inhibitors
AR-V7	AR splice variant 7
BAP	Bone-specific alkaline phosphatase
BMM	Bone metabolic markers
BSI	Bone scan index
cf-DNA	Circulating cell-free DNA
CICP	C-terminal of type-1 collagen pro-peptide
CPS	Combined Positive Score
CSS	Cancer-specific survival
CTC	Circulating tumor cells
ct-DNA	Circulating tumor DNA
CTLA-4	Cytotoxic T-lymphocyte-associated protein 4
CTP	C-telopeptide
CYP17A1	Cytochrome P450 17 alpha-hydroxylase/17,20 lyase
DBD	DNA-binding domain
DHEA	Dehydroepiandrosterone
DHT	Dihydrotestosterone
dMMR	Mismatch repair deficiency
DNA	Deoxyribonucleic acid
dsDNA	Double stranded DNA
ESR	Erythrocyte sedimentation rate
ERG	E26 transformation-specific (ETS)-related gene
FDA	Food and Drug Administration
HRR	Homologous recombination repair
HSD3B1	3-beta-hydroxysteroid dehydrogenase-1
ICI	Immune checkpoint inhibitors
LBD	Ligand-binding domain
LDH	Lactate dehydrogenase
LHRH	Luteinizing hormone-releasing hormone
mCRPC	Metastatic castration-resistant prostate cancer
mHSPC	Metastatic hormone-naïve/sensitive prostate cancer
MMR	Mismatch repair
mRNA	Messenger ribonucleic acid
MSI	Microsatellite instability
NCI	National Cancer Institute
NLR	Neutrophil-to-lymphocyte ratio
NTD	N-terminal domain
NTx	N-telopeptide
ORR	Objective response rate
OS	Overall survival
P1NP	N-terminal propeptide of collagen-1
PARP	Poly (ADP-ribose) polymerase
PD-1	Programmed-death receptor-1
PDL-1	Programmed-death ligand-1
PET/CT	Positron emission tomography/computed tomography
PFS	Progression free survival
PI3K	Phosphatidylinositol 3-kinase
PSA	Prostate-specific antigen
PSADT	PSA doubling time
PSMA	Prostate-specific membrane antigen
PTEN	Phosphatase and tensin homologue
PYD	Pyridinoline
rPFS	Radiographic progression free survival
RT-PCR	Reverse-transcriptase polymerase chain reaction
SLFN11	Schlafen family member-11 DNA/RNA helicase
SOX9	SRY-related HMG box-9
ssDNA	Single-stranded DNA
SUV	Standardized uptake value
tALP	Total alkaline phosphatase
TMB	Tumor mutational burden
^68^Ga	Gallium-68
^89^Sr	Strontium-89
^153^Sm	Samarium-153
^177^Lu	Lutetium-177
